# MRI Manifestations of Primary Intraspinal Synovial Sarcoma: A Case Report

**DOI:** 10.5334/jbsr.3253

**Published:** 2023-09-04

**Authors:** Yujie Peng, Xiangxin Zeng, Yong Du

**Affiliations:** 1 Affiliated Hospital of North Sichuan Medical College, China

**Keywords:** synovial sarcoma (SS), spinal canal, MRI

## Abstract

Synovial sarcomas represent a highly malignant form of mesenchymal tumors. The incidence of synovial sarcomas in the spinal canal is exceptionally rare and has been scarcely reported.

**Teaching Point:**

Comprehending the magnetic resonance imaging manifestations of intraspinal synovial sarcoma is important to prevent misdiagnosis.

## Introduction

Synovial sarcoma (SS) is an infrequent, highly malignant mesenchymal tumor, accounting for approximately 5–10% of all soft tissue sarcomas [1]. The peak incidence of SS occurs between 30 and 40 years of age, with 90% of cases developing before the age of 60. The male to female ratio is approximately 1.2:1 [1]. SS primarily manifests near limb joints, such as the knee and ankle, and occasionally in the head, neck, mediastinum, lungs, and other regions. However, the occurrence of primary SS in the spinal canal is exceedingly rare [2]. Intraspinal synovial sarcomas are scarcely reported in the current literature. This paper aims to present the magnetic resonance imaging (MRI) manifestations of a case involving primary intraspinal synovial sarcoma to enhance the understanding of the disease among radiologists.

## Case History

A 65-year-old woman presented with persistent pain in the right posterolateral lumbosacral region and right lower limb for over a year. The pain was accompanied by continuous burning sensations, fatigue in the right lower limb, and difficulty in walking. However, there were no reports of dizziness, headache, incontinence, or other symptoms. Upon physical examination, grade IV muscle strength was observed in both lower limbs. Warm tactile sensation was absent in the right posterolateral lumbosacral region and could not be perceived above the posterolateral knee of the right lower limb or below the waist. The bilateral Babinski and Hoffman signs were negative. Laboratory examinations yielded no abnormalities. The patient had no significant prior medical history or family history and no evidence of neurofibromatosis.

**Figure 1:**
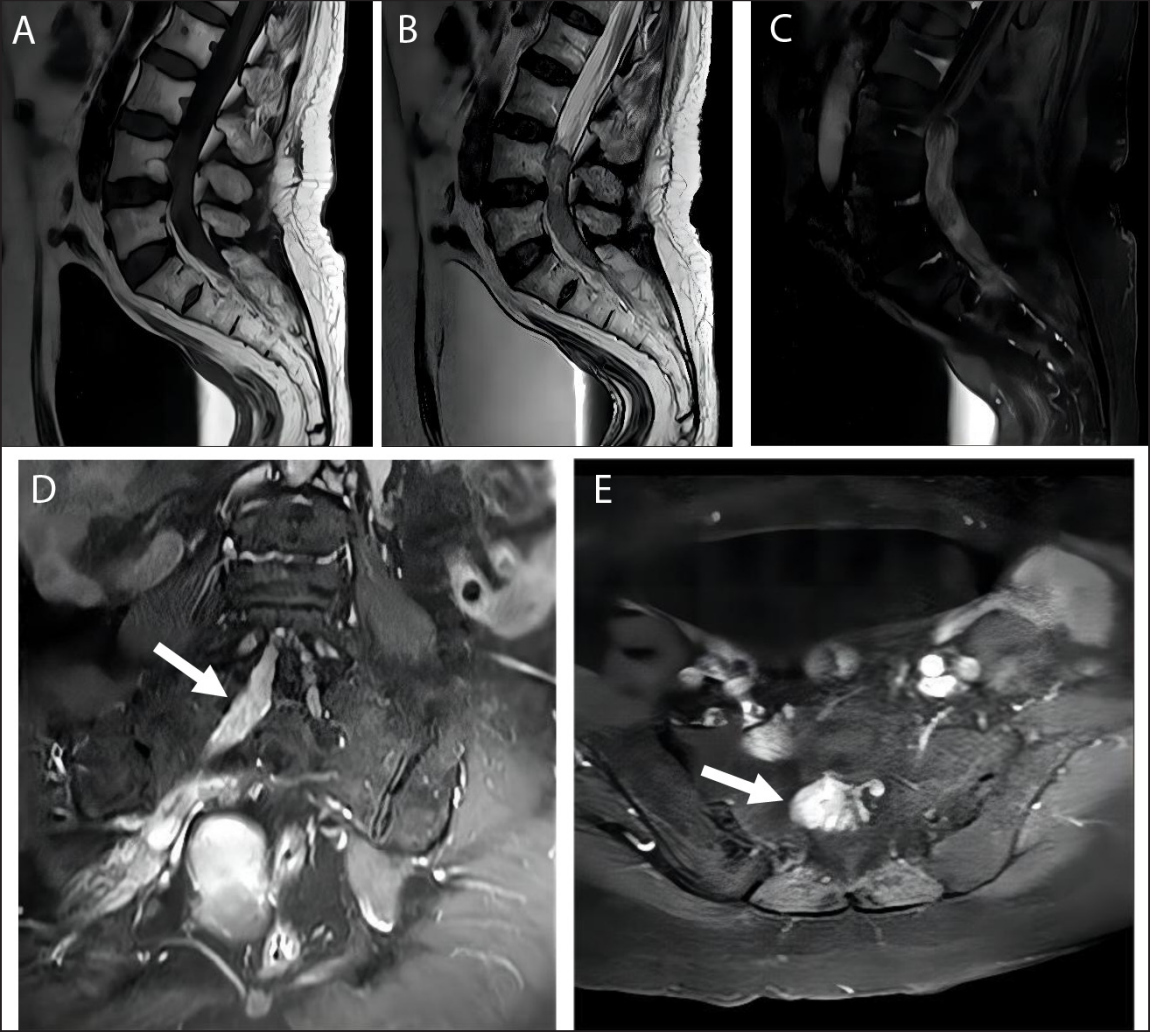
MRI images of SS in spinal canal. T1WI sagittal **(1A)** displays a striped, iso-signal mass within the spinal canal; T2WI sagittal view **(1B)** reveal an iso-signal mass with mixed patchy high signal areas; T1WI enhanced sagittal view **(1C)** shows significant inhomogeneous enhancement; T1WI coronal view **(1D)** and axial view **(1E)** show (arrow) demonstrate that part of the lesion extends outward along the first and second sacral foramen on the right.

MRI revealed a strip-like mass in the spinal canal at the level of L4-S2, measuring 6.5 cm × 1.6 cm in size. The mass exhibited an isosignal on T1WI (Figure 1A), and exhibited an isosignal and a mixed patchy high signal on T2WI (Figure 1B). Contrast-enhanced MRI indicated significant inhomogeneous enhancement of the mass, with compression leading to narrowing of the adjacent bilateral subarachnoid space. Additionally, a portion of the lesion (arrow) extended outward along the first and second sacral foramens on the right (Figure 1C–E).

### Surgery and pathology

**Figure 2:**
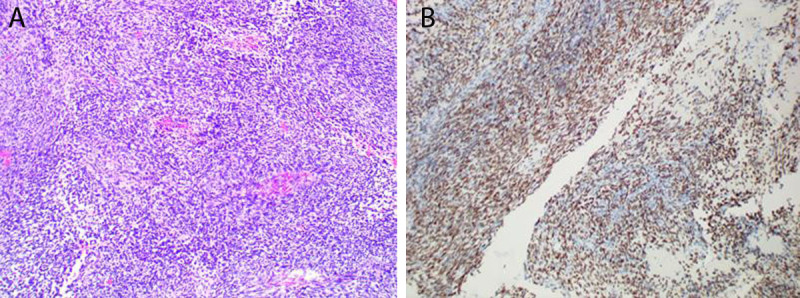
Pathological results. hematoxylin-eosin staining (×100) **(2A)**, Immunohistochemical staining **(2B)**.

The patient underwent intraspinal resection, and during the operation, the lesions exhibited a fish-like appearance, with an abundant blood supply. The lesions closely adhered to the peripheral nerve root, with high dural tension, necessitating the cutting of the dura mater, resulting in the tumor gushing out. Postoperatively, fusiform tumor cells when observed under a light microscope. Immunohistochemistry results were as follows: CK (-), Syn (-), ki-67 (+, 20%), TLE-1 (+), H3k27me3 (+), and S-100 (+); FISH test: SS18-SSX (+, 50%) (Figure 2), suggesting the existence of SS18-SSX gene fusion. The pathological diagnosis was intraspinal synovial sarcoma.

## Comments

The occurrence of primary SS in the spinal canal is exceedingly rare. The imaging findings of intraspinal SS lack distinct specificity. However, MRI plays a crucial role in depicting the tumor’s location, its relationship with surrounding structures, and assessing pathological changes. Typically, MRI manifestations of intraspinal SS exhibit equal or slightly lower signal intensity on T1WI, with focal high signal intensity due to hemorrhage and predominantly high signal intensity on T2WI. Approximately 33% of tumors display typical triple signals, characterized by a mixture of high, equal, and low signal intensities [3]. This variability may be attributed to intratumoral bleeding, old hemorrhage, hemosiderin deposition, or calcification. Notably, the lesions demonstrate progressive enhancement on contrast-enhanced MRI, with the delayed image showing conspicuous uneven enhancement [4]. Computed tomography (CT) imaging often reveals a solid mass with a well-defined boundary and uniform or slightly lower density. Focal calcification may be observed in about 30% of the lesions, and the tumor could exhibit mild enhancement upon contrast-enhanced CT. In addition, the invasive growth of lesions along the first and second sacral foramen, coupled with the presence of corresponding clinical symptoms, indicates a high likelihood of malignant tumors. Therefore, intraspinal SS is easily confused with malignant neurilemmoma, including schwannoma and neurofibroma, with similar signal and inhomogeneous enhancement on MRI, and malignant schwannomas can also be invasive and involve surrounding structures. At present, there are no clear imaging features to distinguish intraspinal SS from other diseases.

## Conclusion

Primary intraspinal synovial sarcoma is a rare malignant tumor with atypical radiological manifestations. On the basis of the imaging features, it is not possible to differentiate a synovial sarcoma from a malignant peripheral nerve sheath tumor, but the degree of extension of the intradural tumor along the peripheral nerve sheaths should raise the suspicion of a sarcoma.
